# Copernicus for urban resilience in Europe

**DOI:** 10.1038/s41598-023-43371-9

**Published:** 2023-09-27

**Authors:** Nektarios Chrysoulakis, David Ludlow, Zina Mitraka, Giorgos Somarakis, Zaheer Khan, Dirk Lauwaet, Hans Hooyberghs, Efrén Feliu, Daniel Navarro, Christian Feigenwinter, Anne Holsten, Tomas Soukup, Mario Dohr, Mattia Marconcini, Birgitte Holt Andersen

**Affiliations:** 1 Foundation for Research and Technology Hellas, Institute of Applied and Computational Mathematics, Remote Sensing Lab, Heraklion, Greece; 2 University of the West of England, Bristol, UK; 3 Flemish Institute for Technological Research (VITO), Mol, Belgium; 4 TECNALIA, Basque Research and Technology Alliance (BRTA), Derio, Spain; 5 Universitaet Basel, Basel, Switzerland; 6 PIK, Potsdam Institut Fuer Klimafolgenforschung, Potsdam, Germany; 7 Bauhaus Earth, Berlin, Germany; 8 Gisat S.R.O., Prague, Czech Republic; 9 GeoVille Informationssysteme Und Datenverarbeitung GMBH, Innsbruck, Austria; 10 DLR, Deutsches Zentrum Für Luft- Und Raumfahrt, Weßling, Germany; 11 CWare Aps, Copenhagen, Denmark

**Keywords:** Climate sciences, Environmental sciences, Environmental social sciences, Natural hazards

## Abstract

The urban community faces a significant obstacle in effectively utilising Earth Observation (EO) intelligence, particularly the Copernicus EO program of the European Union, to address the multifaceted aspects of urban sustainability and bolster urban resilience in the face of climate change challenges. In this context, here we present the efforts of the CURE project, which received funding under the European Union’s Horizon 2020 Research and Innovation Framework Programme, to leverage the Copernicus Core Services (CCS) in supporting urban resilience. CURE provides spatially disaggregated environmental intelligence at a local scale, demonstrating that CCS can facilitate urban planning and management strategies to improve the resilience of cities. With a strong emphasis on stakeholder engagement, CURE has identified eleven cross-cutting applications between CCS that correspond to the major dimensions of urban sustainability and align with user needs. These applications have been integrated into a cloud-based platform known as DIAS (Data and Information Access Services), which is capable of delivering reliable, usable and relevant intelligence to support the development of downstream services towards enhancing resilience planning of cities throughout Europe.

## Introduction

Climate change has become one of the greatest challenges of this century. Cities are integral to the progress of economic and cultural development; however, they are also considered hotspots of climate change, with their susceptibility and exposure to climate-related hazards on the rise^1^. Risks related to urban heat, air pollution, flood, landslides and subsidence are critical for the security and resilience of cities and are further exacerbated by climate change. In addition to these direct effects, climate change has several indirect impacts on cities and their citizens^2–4^. High temperatures also put infrastructure at risk, deforming roads and railways hampering the movement of goods and commuters. These direct and indirect impacts of climate change challenge the environment, economy, quality of life and functionality of cities across Europe as a whole. These impacts are also the driving force for urban resilience planning that seeks to deliver policy objectives as defined in the 2030 Agenda for Sustainable Development, the Paris Agreement and Glasgow Climate Pact, the Sendai Framework for Disaster Risk Reduction, the New Urban Agenda^5^, and the Sixth Assessment Report of the Intergovernmental Panel on Climate Change (IPCC)^6^, as well as at European level in the European Green Deal (European Climate Law), the European Union (EU) Climate Adaptation Strategy, the EU Missions, the New Leipzig Charter, the New European Bauhaus, the 8th Environment Action Programme, and the Covenant of Mayors for Climate and Energy^7–12^.

Resilience planning has therefore become an increasing necessity for cities, particularly in the face of climate change. Urban resilience refers to the [ability of an urban system … to maintain or rapidly return to desired functions in the face of a disturbance, to adapt to change, and to quickly transform systems that limit current or future adaptive capacity]^13^. The urban system is however a fundamentally complex ecosystem of socio-economic and environmental characteristics that are substantially interdependent and interconnected. Accordingly, urban resilience planning interventions seek to effectively manage this urban complexity deploying integrated urban policy strategies that deliver “win–win” policy co-benefits. These policy co-benefits seek to simultaneously secure socio-economic as well as environmental policy goals in a manner that are mutually supportive in frameworks of comprehensive planning strategies.

Here, we focus on the contribution of the CURE (Copernicus for Urban Resilience in Europe) project towards fostering innovation in the field of urban resilience. CURE is funded by the EU Horizon 2020 Research and Innovation Framework Programme and exploits Copernicus space infrastructure to monitor the physical organisation of urban areas, which is central to urban resilience planning. Copernicus is the **E**U Earth Observation (EO) programme that provides accurate, timely and easily accessible information to improve the management of the environment, understand and mitigate the effects of climate change and ensure civil security. Research on spatial pattern detection enables intra-urban analysis across scales, where EO data provide critical intermediate links for building, block, neighbourhood, city and regional scales. By using EO to integrate research across scales, CURE can help to bridge knowledge and solutions that are currently isolated in local planning, regional assessment, national policy and international negotiations.

Critically, city authorities need evidence to help them manage risks and implement suitable strategies for resilience. Mitigation and adaptation actions that enhance the resilience of cities must be based on an integrated assessment and quantification of the drivers of urban transformation, vulnerability of urban citizens and the built-environment, disaster risk and local and global climate change criteria, that secure the required policy co-benefits in a framework of sustainable development. The potential of EO to provide the intelligence on urban form and function required at different spatial and temporal scales is substantial. EO online platforms e.g., the EO Toolkit for Sustainable Cities and Human Settlements, and initiatives, such as the EOs for the Sustainable Development Goals (SDGs) and the Group on EOs (GEO), make significant contributions with global coverage. However, the required tailored, scalable, and context relevant intelligence supporting effective and timely climate action at the local level is frequently unavailable to the urban planning and decision-making community^14^. The potential for integration of EO with other locally derived intelligence is therefore of critical interest in the promotion of evidence-based resilience planning and effective support for the monitoring of progress towards implementation of the SDGs and European Green Deal at the local scale^15,16^.

A major advantage of Copernicus is the ability to support consistent decision-making assessment of across the universe of cities as a basis for integration with local third-party data and methodologies. This capacity and opportunity has been exploited and developed by EU research and innovation projects^17–20^. These projects have prepared the ground for innovative exploitation of Copernicus Core Services (CCS), by both the scientific and urban planning communities, in a wide range of activities and emerging applications in the broader context of urban sustainability and resilience. Moreover, other research and innovation projects, notably RAMSES^21^ and Smarticipate^22^, have specified the framework of user requirements for open governance allied with integrated sustainability assessment, and also emphasised the essential requirements for interoperability of urban governance systems. The medium and long-term exploitation of these research project outcomes target the ability to analyse observations from upcoming satellite missions in order to develop new services assessing the implementation of urban interventions^23,24^, including Nature-Based Solutions (NBS)^25^, monitoring of air quality^26^, climate change mitigation planning at neighbourhood level^21^, and sustainable planning strategies to improve the quality of life in cities^18,27–29^.

In this study, an overview of the outcomes of CURE is given, exploring to what extent CURE provided the means to address EO data under-exploitation in urban planning for sustainable and resilient urbanisation. It has become clear that synergies among different investigation methods (in-situ measurements, modelling and remote sensing) and stronger stakeholders’ involvement are needed in urban applications. Critically, the deployment of cross-cutting integration of CCS, including the Land Monitoring Service (CLMS), the Atmosphere Monitoring Service (CAMS), the Climate Change Service (C3S) and the Emergency Management Service (EMS), to meet the requirement for multidimensional urban resilience solutions, and the attainment of policy co-benefits for all parameters specified, is not yet directly available through the CCS. To meet the requirements for an integrated urban resilience strategy, overcoming the CCS limitations has become a central priority. Such a strategy can be based on cross-cutting applications, accessing intelligence from the identified CCS as well as integrating third-party data (e.g., in-situ observations) and modelling to address the required scale and granularity. In this context, CURE aimed at developing cross-cutting city-scale applications for urban resilience that synergistically exploit the CCS and assess their added value in supporting analyses related to climate change adaptation and mitigation, urban health, as well as economic development. Therefore, the main research question of this study concerns whether, and to what extent, the information derived from the CCS is able to provide reliable, usable and useful intelligence enhancing the resilience planning of European cities? Here, we answer this question by demonstrating and subsequently evaluating the technical operational feasibility of combining the CCS to develop cross-cutting applications in the domain of urban resilience planning for several European cities’ case studies. Finally, we demonstrate how these cross-cutting applications integrated in a Data and Information Access Services (DIAS) based prototype developed by CURE, are easily transferable to other urban areas and capable of providing urban resilience benchmark data for different application fields.

## Materials and methods

The overall CURE approach is shown in Fig. S1, which depicts the cross-cutting process of user dialogues and workshops that were organised in May and June 2020, October 2021 and October 2022, facilitating continuous interaction with the user communities in order to define the stakeholder requirements for CURE application development and evaluate the outcomes. The users included representatives of four front-runner and seven follower CURE cities (Table 1). Furthermore, a survey was carried out to gather more requirements from a variety of stakeholders across EU in May 2020. The survey was sent to stakeholders who have interest in remote sensing applications for environmental thematic exploration and decision-making. The 64 survey respondents had expertise in Geographic Information Systems (GIS), urban planning, data analyst, social science, policy making, environment, climate change, software development, Information Technologies (IT), architecture, remote sensing, water and infrastructure, urban hydrology, strategic spatial planning, policy and smart energy. CURE applications are subsequently developed around two pillars: the first related to the proof of concept of Copernicus cross-cutting applications, by developing and benchmarking applications related to different dimensions of urban sustainability (climate change mitigation and adaptation, healthy cities, energy and economy), based on CCS products (CLMS, CAMS, C3S and EMS); the second concerned the CURE system development, using methods and sample data from each cross-cutting application, as well as the evaluation of its urban resilience potential. In addition to Copernicus core products, third-party data are also used, such as in-situ measurements from networks of meteorological stations, flux towers for direct measurements of heat and Carbon Dioxide (CO_2_) emissions, health profiles and energy consumption, traffic and population data. The development of the applications is implemented in two stages: first, applications are developed for the front-runner cities (Table 1) to examine deployed methods, input data and results; second, the developed applications are replicated for the follower cities (Table 1) to verify their transferability potential. The evaluation and benchmarking of the different approaches are supported by auxiliary spatial datasets, whereas in-situ observations are used to downscale Copernicus-derived city scale atmospheric parameters that are needed to force CURE models, as well as to verify model outputs. The performance of the CURE system is also evaluated and demonstrated in the framework of two demonstration events.

**Table 1:** The CURE cross-cutting applications.

AP	Cross-cutting applications	Berlin	Copenhagen	Sofia	Heraklion	Bristol	Ostrava	Basel	Munich	San Sebastian	Vitoria-Gasteiz
01	Local scale surface temperature dynamics	●	●	●	●	●	●	●	●	●	●
02	Surface urban heat island assessment	●	●	●	●	●	●	●	●	●	●
03	Urban heat emissions monitoring				●			●			
04	Urban CO_2_ emissions monitoring				●			●			
05	Urban flood risk				●		●				
06	Urban subsidence, movements and deformation risk				●		●				
07	Urban air quality			●		●	●				
08	Urban thermal comfort		●	●			●			●	
09	Urban heat storage monitoring				●			●			
10	Nature-based solutions			●						●	
11	Health impacts (socioeconomic perspective)		●	●		●					

They are all developed for at least one “front-runner” city (among Berlin, Copenhagen, Sofia and Heraklion) and evaluated in one or more “follower cities” (among Bristol, Ostrava, Basel, Munich, San-Sebastian and Vitoria-Gasteiz), following stakeholder consultation on each city needs. Stakeholder requirements shaped the development of CURE cross-cutting applications, informed by specifications in urban resilience and spatial planning.

### The CURE system

The CURE system architecture is structured according to a conceptual frame of three principal considerations developed by the H2020 project Smarticipate^22^: (a) open—governance process and decisions that foster citizen engagement improving the quality of decision-making for public institutions; (b) integrated—assessment of urban complexity supporting enhanced decision-making in a framework of interconnected strategic policy objectives where policy co-benefits and “win–win” solutions are sought; and (c) interoperable—that reflects the commonality of the drivers of change at global and pan-European levels that impact cities, supporting the requirement for generic modular systems of urban governance in which smart city governance solutions are applied universally to European cities. The CURE prototype is a cloud-based system developed as Platform as a Service (PaaS) on DIAS. DIAS offers high-performance and stable access to all relevant EO and ancillary data, fast download capacities and direct processing. After a careful platform selection process, it was decided to implement the working horse for processing chains on WEkEO, which is the EU Copernicus DIAS reference service for environmental data, virtual environments for data processing and skilled user support. WEkEO offers a large range of pre-processed Sentinel satellite fleet data, data from Copernicus Services, and additional in-situ data. In total, 235 open datasets are available ranging over many thematic and geographic areas. Besides the data availability, WEkEO offers additionally compute infrastructure in terms of typical cloud virtualised engines. They are arranged along different virtual processing environments, suitable to serve the required distributed processing system. CURE was built as a Front-office on WEkEO and a CCS interface. A relevant data portfolio for the cross-cutting applications was also developed and OGC (Open Geospatial Consortium) OpenSearch was used for the standard service, facilitating the aggregation of results among disparate data providers and collections. It was further extended into a richer semantic-based service using urban resilience ontology as a dedicated transversal service focused on service providers.

### The CURE cross-cutting applications

Eleven specific applications (Fig. S2), reflecting specific urban sustainability dimensions (climate change adaptation and mitigation, healthy cities, energy and economy) are developed across CCS, based on state-of-the-art methods that were developed in past projects. However, in CURE, these methods have been improved and adapted for cloud computing and CCS. Each method is separately given below, explaining how each cross-cutting application makes use of products derived from at least two CCS.

#### AP01: local scale surface temperature dynamics

The Land Surface Temperature (LST) downscaling approach developed by Mitraka et al.^30^ provided the basis for AP01. Atmospheric information from CAMS and surface-cover information from CLMS are used. Existing CLMS LST assumes the land surface to be composed of vegetation and bare soil and delivers LST in 5 × 5 km spatial resolution. When referring to the temperature of cities it is important to have local scale LST (i.e., 100 × 100 m) derived accounting for the urban surface, as well. Here, the downscaling approach is based on a Sentinel 2 and Sentinel 3 synergy, and it is capable of providing surface temperature estimates at local scale at the frequency of Sentinel 3. The method is improved by adapting it to CCS to derive coherent LST estimates across all targeted cities. Surface-cover information in high spatial resolution is used to downscale the low-resolution Thermal Infrared Radiance (TIR) for generating a high-resolution TIR band. Surface information (land cover, vegetation indices and soil moisture), as derived from CLMS, are combined with fractional surface-cover retrievals (Sentinel 2) and spectral libraries^31,32^ to estimate surface emissivity^30,33^. By combining the high-resolution TIR and emissivity with atmospheric information from CAMS in a split window algorithm^34^, local scale LST is estimated. The downscaled LST values are evaluated with *in-situ* outgoing longwave radiation observations in the radiometer source area of flux towers.

#### AP02: surface urban heat island assessment

The study of Surface Urban Heat Island Intensity (SUHII) using EO is usually conducted over selected pixels that are located in the urban and rural regions, separately. However, fixed pixels only reflect part of the SUHII characteristics and solely represent the micro-climate of the local area to which they refer. On the other hand, the different definitions of urban/rural regions make the inter-comparison study of SUHII among different cities particularly challenging. To overcome these limitations, the approach of Li et al.^35^ is employed, allowing the calculation of SUHII (and its temporal dynamics) by exploiting the linear relationship between LST and the Percentage Impervious Surface (PIS) derived from CLMS. Given the LST footprint (provided by AP01), the PIS is regionalised to include information of neighbour pixels within the footprint by means of Kernel Density Estimation. Then, a linear regression function of LST is fitted using the regionalised PIS and SUHII is computed as the regression slope of the fitted function. Ultimately, the outputs of AP02 are estimated SUHII values per city, per targeted temporal interval (e.g., monthly, bi-monthly), whose interpretation enables a relative ranking of cities by their thermal characteristics.

#### AP03: urban heat emissions monitoring

Urban heat emission refers to the heat exchange between the urban surface and the atmosphere, namely the Turbulent Sensible Heat Flux (Q_H_) and it is based on the Aerodynamic Resistance Method (ARM)^36^. Here, the ARM implementation described in Feigenwinter et al.^37^ is further developed and adapted to suit the requirements of CURE end-users. It is based on the surface layer similarity theory where *Q*_*H*_ is calculated as a function of the LST (provided by AP01), the air temperature provided by C3S and the aerodynamic resistance, which is derived by combining surface cover and building height information from CLMS and third-party data. CLMS urban surface information is combined with elevation information and *in-situ* observations to estimate the parameters needed in aerodynamic resistance estimation^37^; the roughness parameters are calculated as per Kent et al.^45^. The method is evaluated using flux tower observations^18,38^.

#### AP04: urban CO_2_ emissions monitoring

The approach for estimating within urban boundaries’ CO_2_ emissions is based on statistical modelling of all individual sources (the four basic sources and sinks in the urban environment, namely: the emissions from fossil fuel combustion by motor vehicles, the emissions from combustion within buildings, the metabolic release of CO_2_ by human respiration and the source/sink contribution by vegetation and soil. The method relies mainly on combining local scale CO_2_ flux observations by Eddy Covariance with geospatial information of urban form parameters through analytic source area modelling^39–41^ and the comparison with literature data for the individual sources. The relationships between traffic frequencies, building morphology, population density and vegetation are determined in an iterative way by comparing modelled emissions with local scale contributions from the respective emission sector in the source area of the CO_2_ flux measurements. Air temperature derived from C3S is used to calculate heating degree days, which are used as an auxiliary parameter for emissions from the building sector where relevant. Emission estimates from human metabolism are based on spatially aggregated population statistics^42^ derived from CLMS products and local census data. Since the vegetation fraction in the footprint of urban flux towers is usually very small, the biogenic contribution in the area of interest is modelled by an empirical approach based on seasonal Normalised Difference Vegetation Index (NDVI) derived from Sentinel-2. The individual contributions of each different CO_2_ emission component are upscaled to the urban boundaries according to the geospatial products used (CLMS, C3S), yielding resulting in mean daily city-wide and then summed to net CO_2_ flux products. The final products are evaluated using independent flux tower measurements and compared with CO_2_ emission products of CAMS.

#### AP05: urban flood risk

The main added value of AP05 relies on the combination of flood exposure and urban asset information. The former is estimated by means of hydrological/hydraulic modelling, which takes into consideration terrain morphology, river network characteristics, and intensity of precipitations. The latter is derived by accounting for both spatial distribution and temporal evolution, based on CLMS and the World Settlement Footprint^43^. The flood-related products developed in the context of Copernicus EMS provide information about location, intensity and optionally evolution of flood hazards. Thus, they represent key input into identification of exposed assets, population and infrastructure and assessment of flood-related risks. Hypothetic inundation extent and depth relevant for selected flood recurrent frequencies (e.g., 50 or 100 years) are modelled based on accurate hydro- meteorological records, land-cover maps and digital terrain model. Here, EMS and CLMS are used to provide flood risk maps, combining flood hazard maps with maps of assets (land cover/use at building block level, buildings, and infrastructure elements). Information on land cover/use, building types and population density and monetary value of built-up areas where available, are used to quantify the vulnerability of exposed assets to floods. AP05 provides information about flood hazard as raster maps representing flood susceptibility, frequency or extent and inundation depth for different flooding scenarios, which can either be used on their own or combined with datasets describing urban areas, to obtain meaningful information about the flood risk exposure of these areas.

#### AP06: urban subsidence, movements and deformation risk

AP06 is based on Kolomaznik and Hlavacova^44^. It is based on interferometric persistent scatterer technique—processing of time series of high or very-high resolution satellite Synthetic Aperture Radar (SAR) imagery which enable detection of up to millimetre displacements and has been demonstrated in number of national^45,46^ and global EMS related activations^47^. Here, the above approaches are adapted to consider the cross-cutting aspects of urban subsidence that benefitted from the combination of EMS, CLMS and C3S services utilised for subsidence risk assessment application, coupling hazard monitoring with up-to-date assets information (land cover/use at building block level, buildings, and grey infrastructure elements) and climate change scenarios. AP06 initially produces information about the subsidence itself, based on multi-temporal Interferometric SAR techniques. Then, it integrates subsidence related products (annual subsidence velocity for each permanent point scatterer inside a pre-defined area of interest) with relevant additional data and information from CLMS to derive meaningful intelligence and understanding about the ongoing subsidence-related processes in the city and surrounding areas. This includes clustering of stable points with subsidence information and spatial overlay analysis with the layers representing the structure and development of the city.

#### AP07: urban air quality

Pollutant concentrations at street-level scale are influenced by regional background concentrations, urban increments due to local industrial and traffic sources, and an additional contribution due to the recirculation in the street canyon. Here, we therefore propose a solution which captures the multi-scale aspect by combining several models into an integrated model chain, the ATMO-Street chain^26^. The final outputs of this service are street-level yearly mean Nitrogen Dioxide (NO_2_) and Particulate Matter (PM2.5) concentrations. The core of the methodology consists of a traffic emission model and a Gaussian dispersion model coupled to a street-canyon module^26^. The model chain has been validated using several measurement campaigns, focusing on spatial patterns and time series alike^26,48,49^. The model chain relies on several input datasets: background atmospheric concentrations, meteorological data, traffic volumes and fleets, industrial emissions, and 3-D building data. Several CCS are used, thereby showcasing the value of the various services in applying the ATMO-street model to analyse air quality at street level where local reliable data is not available. One of the key challenges in modelling street scale is the availability of 3-D building data required as input, which can be provided by CLMS. CAMS re-analysis data are used to provide the background concentrations and meteorological input is taken from the ERA5 re-analysis (C3S). Emission data are combined with higher resolution proxy data to downscale the CAMS emissions to the required resolutions (point sources/surface sources for residential emissions, and line sources for traffic emissions). Two options for the downscaling are considered: (a) OpenStreetMaps (www.openstreetmaps.org) for line sources and the residential emissions of CAMS at their native resolution; (b) a local bottom-up roadmap (with information on the road capacity) and a local dataset describing the location of heaters.

#### AP08: thermal comfort

AP08 is built upon the urban climate model UrbClim^50^, providing urban air temperature, humidity and wind speed at 100 m × 100 m spatial resolution. It provides thermal comfort indicators such as the Wet-Bulb Globe Temperature (WBGT)^51^, aggregating different climatic stressors for the entire urban area. WBGT is currently in use by a number of bodies including the United States (US) and United Kingdom (UK) Military, civil engineers, sports associations and the Australian Bureau of Meteorology^52^. AP08 is based on Liljegren et al.^53^, using the method of Lemke and Kjellstrom^54^ to calculate outdoor WBGT values. It uses CLMS land cover, building data, urban green, imperviousness, soil sealing/imperviousness and NDVI for model configuration and surface specification. Climate forcing data are taken from C3S ERA5 re-analysis data. By combining the standard output of UrbClim model with detailed radiation calculations based on 3D building and vegetation data, AP08 calculates WBGT with a very high spatial resolution^55^.

#### AP09: urban heat storage monitoring

An updated version of the Objective Hysteresis Model that has been recently released^56^ is the basis of AP09: the hysteresis effect on energy flux storage indicates how quickly the urban surface responds to the input of energy and its association with the diurnal evolution of the boundary layer, varying according to latitude, cloud cover, soil characteristics, wetness and vegetation cover. Therefore, AP09 calculates the heat storage as a function of the net all-wave radiation, the rate of change of the net all-wave radiation at the surface, the different surface-cover fractions and three coefficients associated to the response of the surface cover due to the energy input. The first coefficient indicates the intensity of the relationship between the stored energy flux and the net radiation. The second quantifies the magnitude of hysteresis. The third is an intercept term and indicates the extent to which a negative storage energy flux occurs before the net radiation becoming negative. The net radiation is estimated using C3S products based on Chrysoulakis^57^. The LST provided by AP01 is used to estimate the outgoing longwave radiation, whereas the outgoing shortwave radiation is calculated at local scale by combining Sentinel 2 reflectance with lower resolution albedo products^58^. Moreover, the above coefficients are determined from CLMS, based on the land cover as per Grimmond and Oke^59^, whereas the rate of change of the net all-wave radiation is calculated as per Xu et al.^60^, based on CAMS products and *in-situ* air temperature observations. The method is evaluated based on cross-comparisons with respective products based on different approaches^18^.

#### AP10: nature-based solutions

AP10 estimates the potential for green coverage at rooftop level by identifying suitable locations for green roof deployment and supporting decision-making towards broader sustainable urban development. The application allows the calculation of two complementary results: the maximum green roof potential and a roof prioritisation score. To obtain the maximum potential for green roofs, an algorithm is applied to obtain the surface area of each building that does not exceed a certain slope threshold, since flat roofs are more suitable for accommodating vegetation. In addition, those buildings are selected which, due to their structural characteristics, can support the loads associated with green roofs by the year of construction of the building. The prioritisation of the installation of green roofs is then calculated based on the maximum potential and criteria for the area in which the building is located such as NDVI, Imperviousness from CLMS and LST from AP01. The prioritisation is flexible and allows each city to set the weights of the criteria used according to local needs. To monitor the impact on Thermal Comfort, AP08 was implemented in specific case studies, developing several green roofs scenarios and its related impacts and effectiveness.

#### AP11: health impacts (socio-economic perspective)

The main outputs of AP11 are health effects related to air pollution, the emission sources and economic costs attributed to air pollution exposure. The model in the application uses the air quality maps from AP07 coupled to the Evaluation of Air Pollution model^61,62^, which uses an impact-pathway approach to assess health impacts which in this model includes; acute deaths (from short and long term exposure), asthma and bronchitis in adults and children, sick days, hospital admissions for respiratory and cardiovascular diseases, lung cancer as well as years of lost life and total amount of deaths as well as related health costs of exposure to air pollution. The health effects and related health costs are calculated for the total air pollution of the cities. The model can estimate how much of total air pollution originates from local sources and how much originates from sources outside of the city, and thereby the related health costs.

## Results

**Figure 1:**
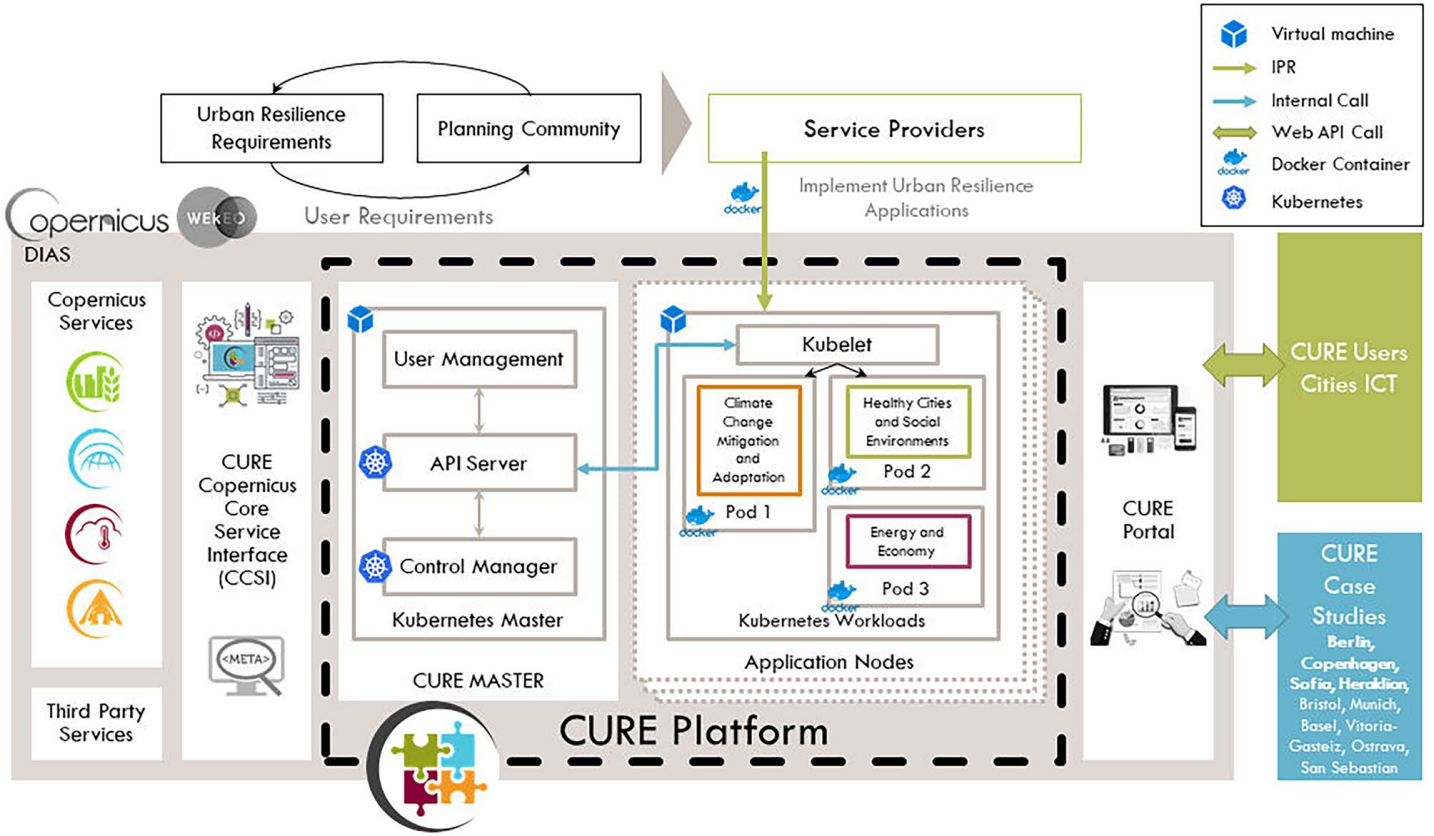
Baseline design of the CURE system. It includes the set-up of hardware components, such as processing units and storage, as well as the software design and interaction between those components, the information provided by the platform (Information as a Service—InaaS) and the deployed applications in a cloud (WEkEO—DIAS) environment. The CURE Prototype is built on two sub services, (i) the CURE MASTER and (ii) the Application Nodes. The CURE Master monitors and orchestrates tasks (Airflow) in the Application Nodes based on their execution state and process chain; offers an Application Programming Interface (API) based access; as well as Graphical User Interface (RESTful APIs well-defined machine-to-machine communication over the web) for the submission, scheduling and monitoring of individual jobs.

The CURE system (Fig. 1) is cloud-based, developed on the DIAS (WEkEO) PaaS. The major advantage of cloud processing solutions is that no expensive purchase, setup and maintenance of hardware and software infrastructure is required, but a rapid and fully automatic provision of all required resources is guaranteed by the cloud platform. Other service providers can access the workflows stored on the platform and apply these to the data hosted on the platform. That allows fully independent, distributed production by different service providers on the one hand, while on the other hand guaranteeing workflows to be consistently applied by partners along working units. As a result, it provides homogenous outputs, which is a key requirement for reproducibility of the processing chains. The system is accessed from the CURE portal (http://portal.cure-copernicus.eu), a web-based application that allows the user to interact with CURE system (Fig. S3) on WEkEO. One part of the portal is based on the concept of storyline to underline the added value of CURE products for user communities. Storylines present the CURE applications, or their combination represented by a set of interactive maps, graphs, charts, tables and other elements used to demonstrate the product’s value and their benefits for the user. The other part of the portal concern user registrations, offering limited access for registered users to generate their own products by using certain CURE applications.

The CURE cross-cutting applications and the relevant cities where they are developed and evaluated in the project are shown in Table 1. Stakeholder requirements shaped the development of CURE cross-cutting applications, informed by specifications in urban resilience and spatial planning***.*** In detail, the CoReS method^63^, applied to support stakeholder engagement and requirements gathering processes, supported the development of an understanding of different user expectations of Copernicus-based data and identified commonalities essential to the development of generic products applied to other cities. City stakeholder requirements were derived in relation to urban planning decision-making delivering sustainability policy targets, concerning climate change, urban health, economic development, etc. This planning requirement is specified according to a variety of policy strategies including climate mitigation and adaptation actions that enhance the resilience of cities and support the definition of transition pathways to sustainable and carbon neutral cities.

The following sections will provide insights into the specific products delivered by the CURE cross-cutting applications. The applications are all developed for at least one “front-runner” city (among Berlin, Copenhagen, Sofia and Heraklion) and evaluated in one or more “follower cities” (among Bristol, Ostrava, Basel, Munich, San-Sebastian and Vitoria-Gasteiz). However, to best illustrate the specifics and characteristics of the data of each application, the results are presented for selected cities and time frames.

**Figure 2:**
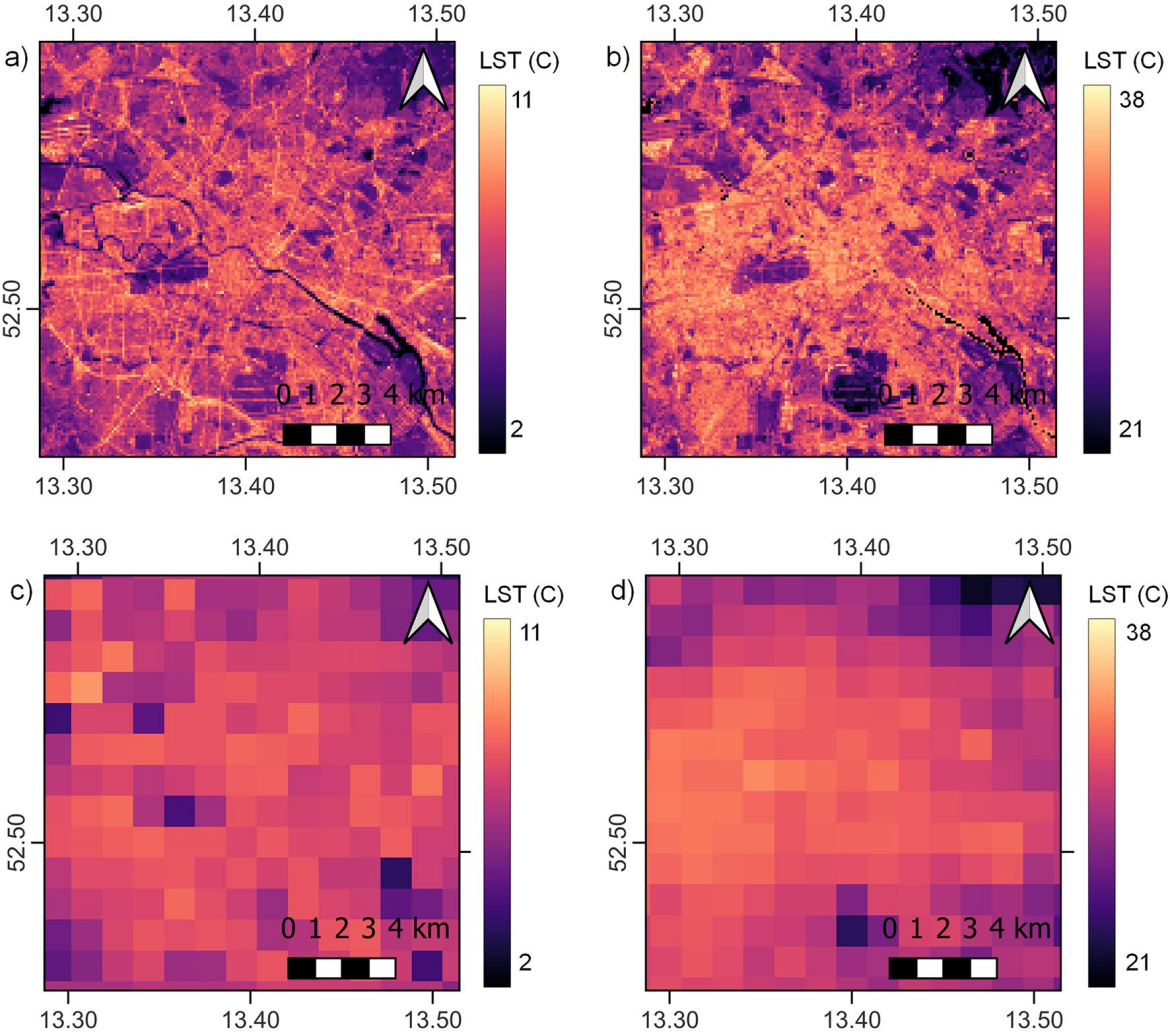
Surface temperature maps for Berlin as resulted by AP01. (**a**) Daytime (30 May 2019, at 11.26 local time); and (**b**) night-time (16 April 2019, 22.32 local time) urban surface temperature maps at 100 × 100 m spatial resolution for the city of Berlin and (**c**,**d**) the respective 1 × 1 km spatial resolution products. Maps created with QGIS software, version 3.16 (www.qgis.org).

CURE Application 1 (AP01) products are detailed day-night LST maps (see Fig. 2, where examples are given for Berlin). The level of detail emerging in the 100 × 100 m spatial resolution products (Fig. 2a and b) is evident, particularly compared to the 1 × 1 km products (Fig. 2c and d). In the daytime product (Fig. 2a) for May 2019, mid-day (11.26 local time) individual temperatures are observed for different surface covers, i.e., paved surfaces including the road network, parking lots and airport runways which exhibit higher values than urban green spaces, reaching up to 37 °C. The night-time product (Fig. 2b) corresponds to mid-April 2019, a couple of hours after sunset. The slow release of heat in the urban areas is evident, which remain hot compared to the surroundings after sunset. Surface temperature maps like this can directly provide quantitative information for the impact interventions, where for example urban trees can affect a remarkable reduction in surface temperature.

SUHII temporal profiles are produced from AP02. They clearly distinguish between different thermal properties, as well as their annual dynamics. Results for “front-runner” cities (Table 1) presented in Fig. S4 are derived for bi-monthly LST data for the years 2018 and 2019. High SUHII values indicate a pronounced heating of the city core as opposed to its rural surroundings. Values around zero indicate no effect and negative values indicate a cooler city than its surroundings. Noteworthy in addition to the annual profiles are the diurnal variations, which are complementary in understanding urban thermal properties. Furthermore, our analysis has revealed a sensitivity of SUHII to the definition of the urban area, based on SUHII estimation using variably sized buffers around the city centres, in line with past studies^64^.

Figure S5 presents a daytime Q_H_ map for the “front-runner” city Heraklion as created by AP03. This clearly shows lower values in the city centre, the relevant mechanism explained in detail by Parlow^64^. A comparison of measured Q_H_ with weighted modelled fluxes in the footprint of the flux towers showed that measured *Q*_*H*_ are generally higher for all case study cities. Beyond the known drawbacks of the ARM used to estimated Q_H_, analysed by Chrysoulakis et al.^18^, several reasons may lead to these differences, as discussed in Feigenwinter et al.^37^: The uncertainty inherent to Eddy Covariance technique that is used to measure *Q*_*H*_ is in the range of 10%, the representativeness of flux tower measurements in urban environments is reduced compared to rural areas due to the heterogeneity of urban neighbourhoods and there are large (inherent) variations in Eddy Covariance measurements between the averaging intervals which additionally increase the uncertainty for the time of the satellite overpass.

**Figure 3:**
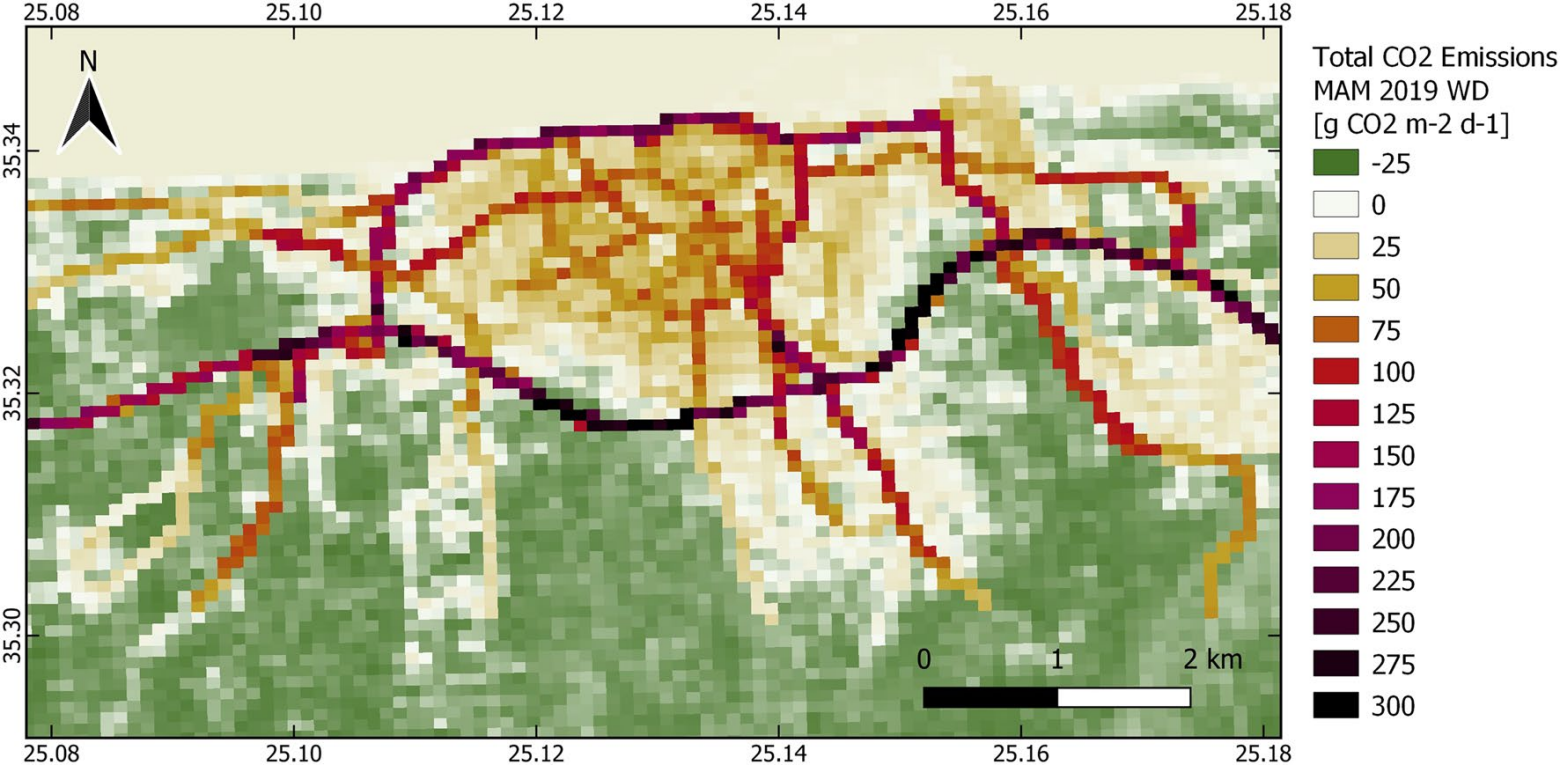
Spatial distribution of mean daily CO_2_ emissions, in g CO_2_ m^−2^ d^−1^, for the “front-runner” city Heraklion, for Working Days in the spring season (March–April-May) in 2019. Highest emissions are linked to traffic (road network) and densely built-up areas (buildings, human metabolism). Vegetated areas act as a sink for CO_2_ during the vegetation period. Map created with QGIS software, version 3.16 (www.qgis.org).

As a product of AP04, the mean daily CO_2_ emissions for the “front-runner” city Heraklion, for the spring season in 2019 is shown in Fig. 3. Highest emissions are linked to traffic and densely built-up areas. The product is based on seasonal mean diurnal fluxes and the corresponding averaged footprints, i.e., the footprint climatology. The mean diurnal course of CO_2_ fluxes for the meteorological seasons winter, spring, summer and autumn for the years 2018 and 2019 has been analysed. The bi-modal shape indicates that traffic (rush hours) is the main driver of CO_2_ emissions in the footprint of the flux tower located at Heraklion centre. Flux tower measurements also revealed that heating in winter plays a minor role in Heraklion (but not in the “follower-city” Basel; Table 1), since no increase in the mean daily total CO_2_ emissions is observed. On the other hand, no significant signal from vegetation was observed in Heraklion city centre, because this would exhibit lower emissions due to photosynthesis in the vegetation period.

**Figure 4:**
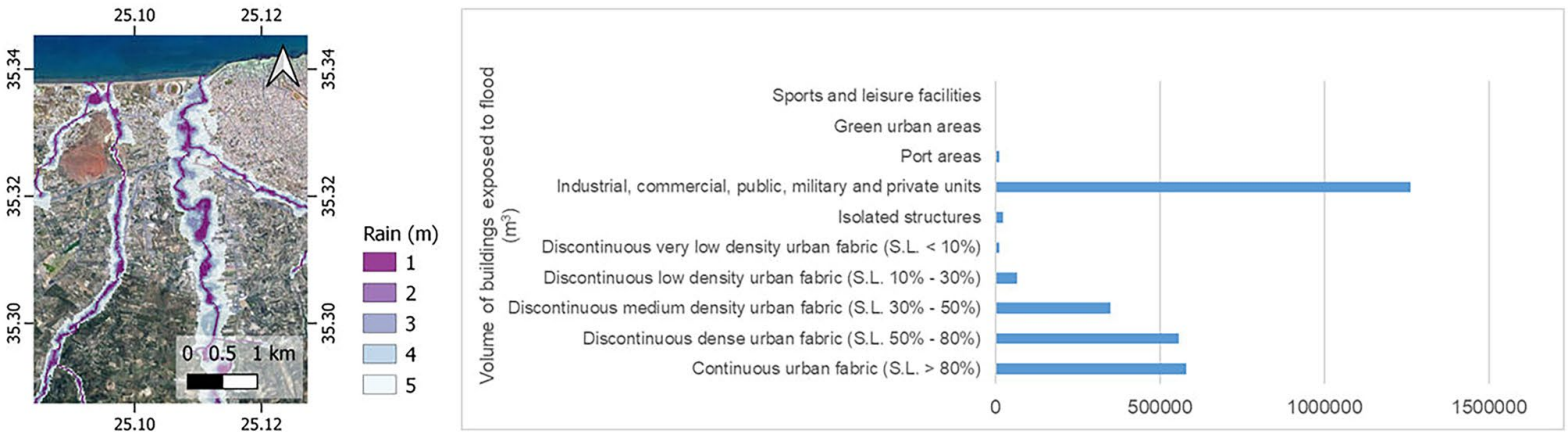
Simple vulnerability analysis for the city of Heraklion: exposure of buildings to flood hazard by land use type (Urban Atlas categories) of the building block. Map created with QGIS software, version 3.16 (www.qgis.org) and graph with Microsoft Excel 2016.

AP05 has initially generated results for different flooding scenarios, corresponding to different intensities of rainfall, which can then be compared by the user. The user can, via the CURE portal, modify this parameter to generate flood masks for his own scenarios. Information about (potential) flood intensity is then combined with information about land use, in particular about distribution and typology of urban areas. Maps of flood susceptibility or frequency and estimations of inundation extent and depth for different flood intensities in the future are integrated with CLMS Urban Atlas data to obtain information about flood hazard for urban areas. Zonal statistics are applied to evaluate flood risk level for each particular land use block. AP05 therefore derives raster maps presenting the distribution and levels of flood hazard in an area. An example of such analysis, calculating the number, area and volume of buildings endangered by flood hazard in the city of Heraklion in total and by the respective land use type is shown in Fig. 4. The hazard is estimated using the high above nearest drainage index approach, where the level of hazard depends mostly on distance to nearest drainage and local terrain properties. Then the hazard to urban assets is evaluated by overlaying the raster layer of flood hazard distribution with delineation of the urban assets of interest. The level of flood hazard is quantified for each analytical unit (building block or single building). Next, the vulnerability of buildings exposed to flood hazard is assessed according to criteria, including the age of building, height of building, construction material, type of building use etc. Consideration of all these aspects can help to evaluate the vulnerability of the building exposed to flood hazard. Moreover, an estimation of maximum cost in case of flood event can be made at the city level or in any given sub-city area.

AP06 results of the analysis for the “follower city” of Ostrava (Table 1) are presented in Fig. S6. The focus here is on an undermined area (as a residual of coal mining activity in the Moravian-Silesian Region). This area is called Poho and it is intended for significant (re)development in the near future, and accordingly, the assessment of subsidence-related hazard is of crucial importance. Furthermore, the surface faulting hazard in the area is assessed to identify not only subsidence on permanent point scatterers or their clusters, but more importantly linear surface faulting, which represents the prime hazard related to subsidence processes, with potential high probability for damage to building or infrastructure.

**Figure 5:**
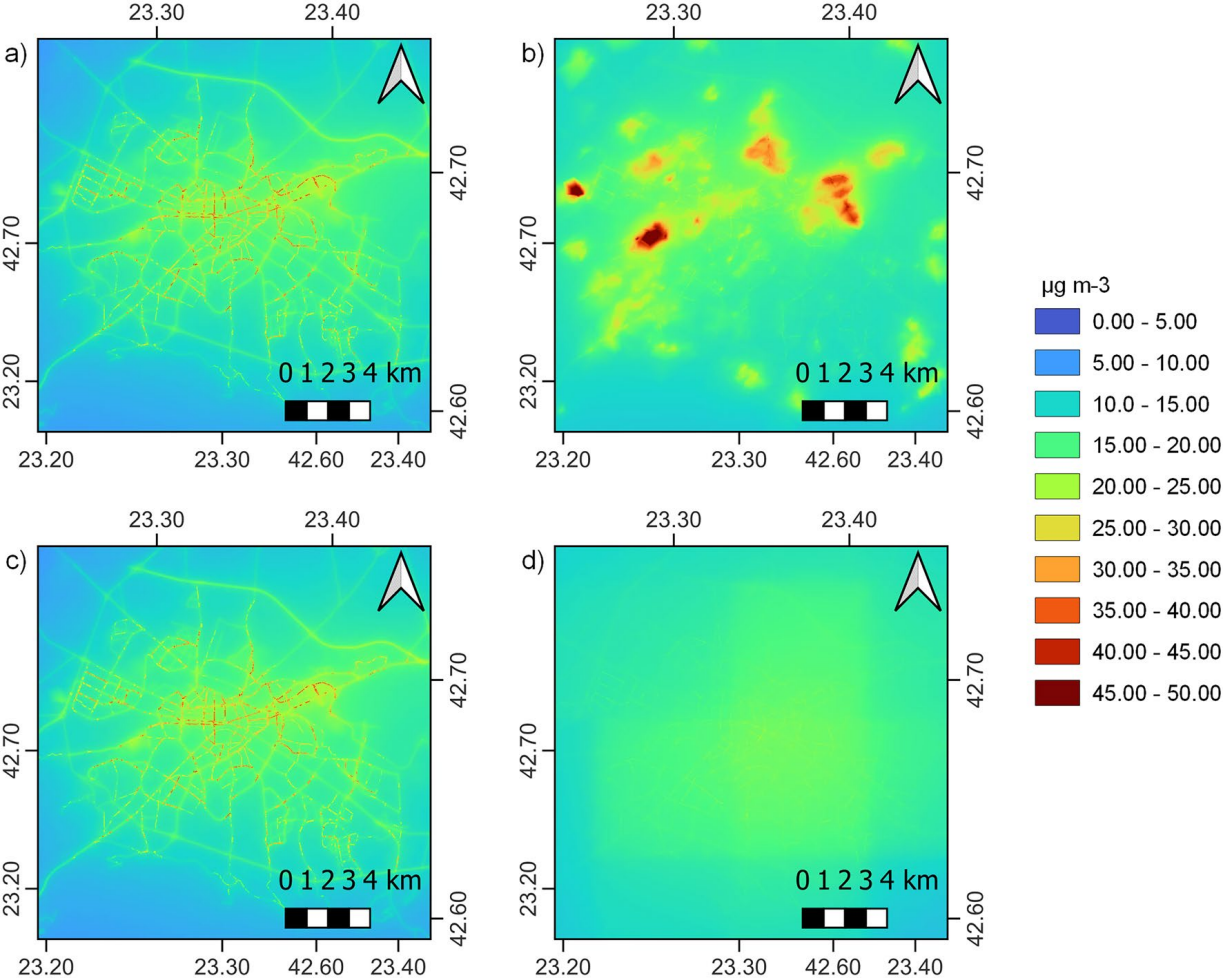
Total annual mean concentrations in μg/m^3^ for the centre of Sofia city: NO_2_ (left column) and PM2.5 (right column). The maps in the bottom row are produced using Copernicus data only, whereas the maps in the top row are produced using downscaled emissions combined with local data. Maps created with QGIS software, version 3.16 (www.qgis.org).

The primary outputs of AP07 are air quality maps describing the yearly mean concentrations of NO_2_ and PM2.5 with a resolution of 10 × 10 m. Figure 5 shows results for the “front runner” city of Sofia. The maps highlight how urban air quality varies significantly over short distances. For NO_2_, the highest concentrations are observed near the major roads, especially “street canyons” in the city centre. The highest concentrations for PM2.5 are, on the other hand, observed in the neighbourhoods with an abundance of residential emissions, especially evident when Copernicus data are supplemented with local data. This type of map allows stakeholders to identify the neighbourhoods and districts with the worst air quality, and to decide in an informed manner for which areas action should be prioritised. Moreover, they can be used for compliance checking with regard to the EU Ambient Air Quality Directive and the more stringent updated World Health Organisation (WHO) guidelines. A second output of AP07 concerns the sector contribution. For each of the sectors explicitly considered in the local modelling (road traffic, industry, power plants and residential heating), the approximate contribution of the individual sector to the total concentration is also provided. Our analysis for Sofia revealed that the urban traffic emissions contributed a significant fraction to the total concentrations, whereas the urban power plant and industrial emissions are responsible for only a negligible fraction of the total pollution. Approximately a third of the pollution at this specific location is emitted outside the domain, or by sectors that are not explicitly considered in the modelling.

**Figure 6:**
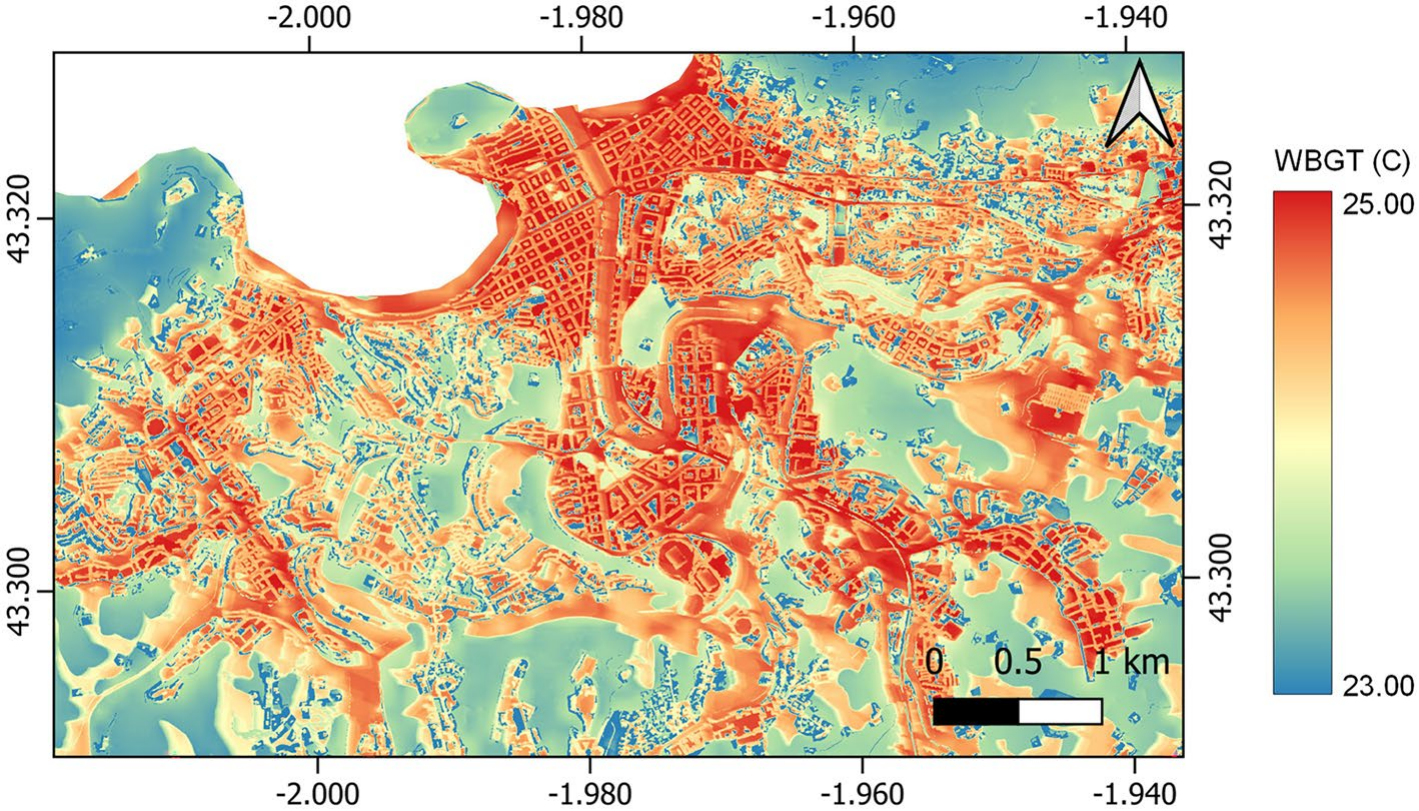
Daily mean WBGT in San Sebastian for 23 July 2019. This map shows a time-average of the heat stress situation and takes also the night-time into account, when the urban heat island is at its strongest. The variability in the WBGT temperatures is typically around 2 to 4 °C. Forested areas are the coolest locations on the map and the bigger they are the larger the cooling effect. Water areas show less cooling in these maps, as they often keep a high temperature during the night, enhancing the urban heat island problem. Impervious areas show, as expected, the highest WBGT values. Map created with QGIS software, version 3.16 (www.qgis.org).

AP08 assesses thermal comfort on the basis of WBGT maps. An example of a WBGT map for San Sebastian, on a typical hot summer day, is shown in Fig. 6. It allows stakeholders to identify hotspots and give them insight in the local variation of heat stress with a high level of spatial detail. The CURE Portal allows users to modify the input land cover map and upload different land use scenarios from which new WBGT maps will be calculated instantly. As a result, users are able to assess the effectiveness of e.g., green–blue adaptation measures and justify their adaptation strategies, in regard to their climate-friendliness. Also, they are enabled to assess the impact of specific intentions in respect to local thermal comfort conditions in the city, which can help them to evaluate alternative solutions from an environmental point of view.

Figure S7 presents the daytime net change in heat storage (ΔQs) in the urban canopy map for the city of Heraklion, as generated by AP09. It is obvious that large amounts of energy are stored within the urban structure of Heraklion during daytime, as expected: the energy from solar radiation is trapped inside the city and it is stored particularly in buildings with an increased rate ranging from 350 to 500 W m^−2^, while in the surrounding rural areas energy is stored in a much lower rate (lower than 200 W m^−2^). The opposite phenomenon occurs during night-time in this example: around midnight the city has released most of the available energy stored during daytime, and subsequently releases at lower rates (around 30–50 W m^−2^). Results are in accordance with previous studies conducted in the same area, based on different approaches^18^.

**Figure 7:**
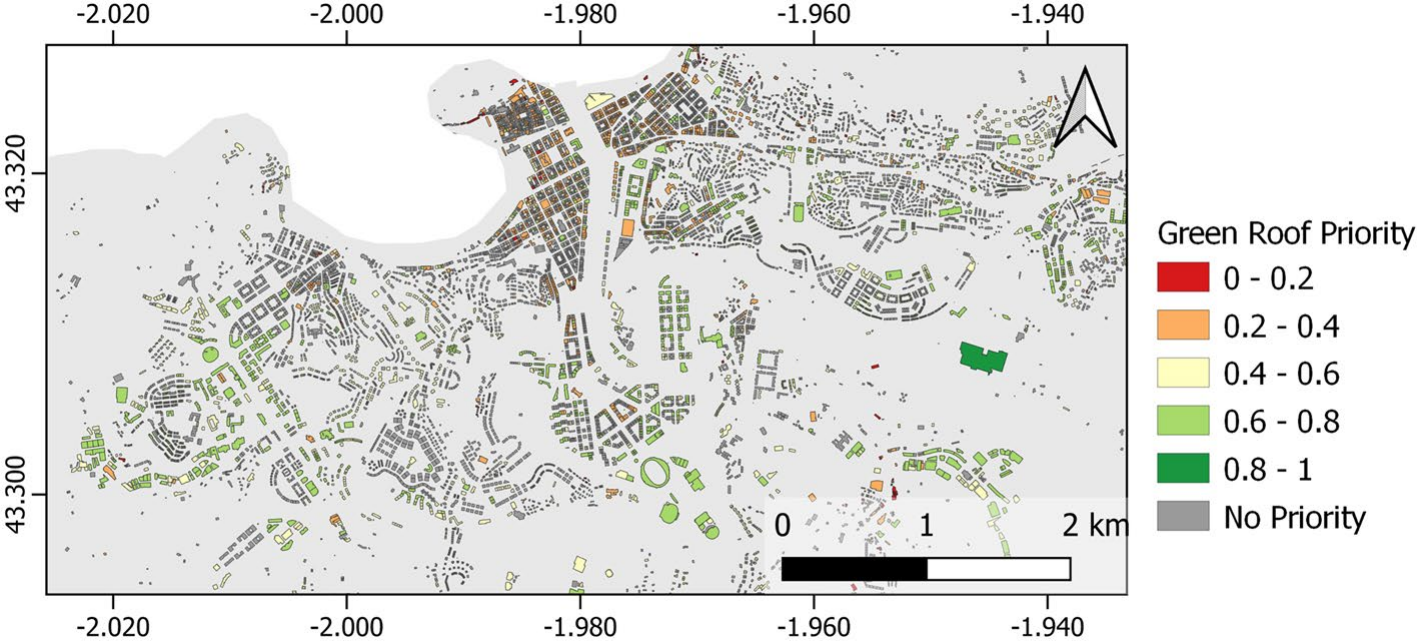
Green roofs priority in San Sebastian. It is obvious that the roofs, which obtain a higher prioritisation, are located in the peripheral areas of the city, which correspond to buildings with a larger roof surface, generally flatter roofs and of lesser age. On the other hand, the zone criteria allow differences to be established in those areas where the LST and imperviousness is high and the NDVI is low. These prioritised zones, therefore, constitute useful information for managing the different risks faced by the city; however, it should be borne in mind that these are not absolute priority values and that they can be modified by decision-makers according to the problems they need to address. Map created with QGIS software, version 3.16 (www.qgis.org).

AP10 estimates the potential for green coverage at rooftop level by identifying suitable locations for green roof deployment, supporting decision-making for sustainable urban development. An example for the city of San Sebastian is shown in Fig. 7. First, a Digital Surface Model (DSM) was used to filter out roofs with slope below a certain threshold and discard buildings, according to construction year, unable to support the structural loads of a green roof, subsequently obtaining the maximum green roof potential in square metres for each building. Buildings are then prioritised according to the previously calculated maximum green roof potential, the year of construction of the building, and the values of the area in which the building is located for LST, imperviousness and NDVI, obtaining a value from 0 to 1, with 1 being the highest priority. Prioritisations can be modified via the CURE Portal on the basis of weighted combinations of criteria that can be specified depending on the city characteristics and the user preferences.

AP11 models the health and economic costs of air pollution in cities. Results for the “front runner” city of Sofia are presented in Fig. S8. Air quality maps produced by AP07 are used (yearly mean concentrations of NO_2_ and PM2.5 for the year 2018), grouped according to sector contribution (road traffic, industry, power plants and residential heating). Using these results, stakeholders can identify key sectors for which measures can be prioritised. The sector contribution also provides a rough estimate for the (theoretical) maximal pollution reduction due to local measures for the sector under consideration. For the city of Sofia calculations are added on the health impacts and related external costs based on information about the sources of pollution and their location, the dispersion of air pollution, as well as exposure of the population, the dose–response relationship between exposure and health effects, and the valuation of health effects, also referred to as external costs related to health effects from air pollution.

The results described above show the proof of concept of Copernicus cross-cutting applications, related to different dimensions of urban sustainability (climate change mitigation and adaptation, healthy cities, energy and economy), based on CCS products. Example analysis results were presented for different cities, showcasing the applicability of CURE methods in various European cities.

## Discussion

The contribution of CURE is mainly focussed on: a user-defined online platform combining CCS to support urban resilience planning; uniform data for large samples of urban areas both within regions and across regions in Europe; consistent measurements across European cities, including synergies between Copernicus core products and third-party data; various approaches and models for better information on urban form and function at different spatial and temporal scales; assimilation of users’ knowledge with technical data and benchmarking; fostering innovation. The innovation potential of CURE lies within the exploitation of the Copernicus offer in the domain of urban resilience, by developing cross-cutting applications combining products from CLMS, CAMS, C3S and EMS with third-party data. CURE has gone beyond the state-of-the-art by adapting and improving EO-based methods to estimate urban parameters at appropriate spatial and temporal resolutions for urban planning processes, using CCS and Sentinels products, specifying and analysing the associated uncertainties and evaluating them using in-situ observations. More specifically CURE:

- Improved the LST downscaling approach for local-scale observations developed by Mitraka et al.^30^ and adapted it to use urban surface information derived from CLMS and atmospheric information derived from CAMS;
- Adapted the ARM^36^ to retrieve heat emissions, incorporating downscaled LST and surface information from CLMS for the aerodynamic parameterisation and air temperature from C3S;
- Retrieved CO_2_ emissions within city boundaries at neighbourhood scale, combining local scale CO_2_ flux measurements with surface parameterisation based on CLMS products and scaled up according to the associated land cover/use type (CLMS) and environmental controls derived from CAMS and C3S;
- Retrieved heat storage in buildings at local scale, using urban surface parameterisation based on CLMS products and the net parameterisation based on CAMS products;
- Improved the approach of Li et al.^35^ for assessing SUHII by exploiting imperviousness and LST estimations generated using inputs from CLMS and CAMS products;
- Improved urban thermal comfort assessment^50^, based on CLMS data on urban land cover, soil sealing, vegetation and building data, as well as on C3S ERA5 re-analysis data;
- Evaluated the impact of NBS in urban environment using urban surface and vegetation information derived from CLMS and Sentinel 2 and combined temperature variation parameters derived from CAMS and C3S with CURE LST and thermal comfort products as well as third-party data determining the green roof potential;
- Applied building data from CLMS to the ATMO-Street modelling chain^26^ to enable the EU-wide inclusion of street canyons when modelling NO_2_ concentrations;
- Updated the cross-cutting potential of mapping urban flood risk by combining EMS with CLMS data to assess the vulnerability of exposed assets and integrate risk financing schemes to buffer flood economic impacts;
- Improved the cross-cutting potential for urban subsidence, movements and deformation risk service by coupling hazard monitoring with asset information (i.e., combined use of EMS, CLMS and potentially C3S services) to assess threats and vulnerabilities of city infrastructure.

Responding to the key research question “whether and to what extent the information derived from the CCS is able to provide reliable, usable and useful intelligence enhancing the resilience planning of European cities”, the overall CURE findings are summarised as below:

### Integrated urban planning and climate monitoring solutions

The Copernicus-based products can be exploited by urban planners and local decision-makers to identify, not only urban areas (hotspots) with intense resilience issues (e.g., exposed communities and infrastructures) that should be addressed but also applicable solutions (e.g., NBS) moderating these issues in these areas. For example, urban overheating is one of the most important parameters in climate monitoring and in response the AP01 monitors the spatio-temporal behaviour of cities’ surface temperature. Additional heat related CURE applications provide frequent local scale surface temperature estimations for cities related to surface urban heat island (AP02), urban heat emissions (AP03), thermal comfort (AP08) and heat storage (AP09). These applications assist urban planners to optimise their adaption strategies with regard to heat stress, and sustainable development optimisation. Combined with other CURE applications, benefits related to key resilience challenges such as climate change mitigation with CO_2_ emissions reduction (AP04) and flooding mitigation (AP05) are also created. The multiscale aspect of flood risk assessment provides a comprehensive picture of the main elements of the risk equation: hazard, vulnerability, exposure and resilience capacity. Additionally, AP06 couples hazard monitoring for subsidence or deformation risk with up-to-date assets information (land cover/use at building block level). Such accurate assessment of threats and identification of vulnerabilities is critical for urban planners to understand and manage risks. Integrated urban planning solutions, promoted via the CURE applications also include NBS (AP10), that generate increased understanding of a wide range of socio-economic and environmental co-benefits arising from, for instance, assessment of the green roof potential and performance of individual buildings for energy conservation with co-benefits of city scale water run-off regulation, thermal comfort, air quality improvement, and contribution to urban heat island moderation. CURE also addresses the critical issue of urban air quality (AP07), which in combination with the specific cross-cutting health impacts application (AP11) support the creation of healthy cities.

### CURE as copernicus urban hub for end-users and stakeholders

Based on the above, CURE is the first research project bringing together all urban-related services from the Copernicus Entrusted Entities. CURE has therefore a unique opportunity to become—or at least to contribute to the development and delivery of—the Copernicus Urban Development Hub, by offering a comprehensive and modular architectural frame defining a robust platform to access Copernicus enabled CURE solutions. The CURE Copernicus Urban Hub conceptual frame is based on specified urban planning end-user information and intelligence requirements supporting complex decision-making process, which generate Copernicus-enabled solutions. Specifically, the Copernicus-based products (heat maps, risk indices, etc.) can be exploited by urban planners to identify, not only urban areas (hotspots) with intense resilience issues (e.g., exposed communities and infrastructures) that should be addressed, but also applicable alternative solutions (e.g., NBS) moderating these issues in these areas. Consequently, these products can also support decision-makers to define the areas of interest and utilise the most appropriate interventions. CURE furthermore identified commonality of user requirements that is critical to the development of generic products applied to European cities to support wider market replicability. It offers a balance between harmonised solutions (including temporally and spatially consistent information) across various cities as well as tailor-made solutions to fill specific needs i.e., involving data collected from the cities, or developing products on demand covering specific requests. Accordingly, the CURE modular approach provides the basis on which common harmonised solutions act as a framework for specific local requirements implementation.

CURE prototype applications have proved the usefulness and viability of combining crosscutting CCS products with local third-party data. The value of these results lies in the applications themselves, allowing transferability and replicability to other cities. Having CCS products as the main source running the applications, ensures data maintenance given CCS is a well-established and consolidated European program, which offers recurrent updates of data that can be used to run again the analysis, wherever it has been already applied, or for implementing the applications in new cities, where CCS data is available. Nevertheless, there are remaining challenges related to interoperability of CCS data and how some applications can be applied with heterogeneous quality and formats of local third-party data depending on the local context, in which they are applied.

### Decision-making across scales

A key to effective planning is the generation of reliable, up-to-date and area-wide information on the urban area supporting decision-making, guiding urban policy making and implementation, and informing and engaging all citizens in the delivery of sustainable urban development^17^. By using EO to integrate policy silos across scales, CURE can help to promote integrated policy solutions and the delivery of the policy co-benefits essential for resilience planning. The significance of this functionality is evident as an ever-growing number of European cities are putting environmental sustainability at the core of their urban development strategies e.g., Covenant of Mayors for Climate and Energy; EU Mission “100 climate-neutral and smart cities by 2030”. Consequently, local and regional authorities are seeking tools and methods, such as the EO-based approach of CURE, that streamline environmental data collection and management and facilitate the exchange of information and best practice at national, regional and local levels. CURE is able to meet these requirements as it provides products at different scales, facilitating comprehension of the underlying mechanisms that drive urban environmental problems. Decision-making across scales capabilities thereby supports the identification of priorities for policy intervention at city and regional levels, at the same time permitting comparability and benchmarking between cities in a pan-European perspective deploying the same tools and criteria for decision-making processes.

### CURE Toolkit for policy support

Smart decisions in a complex and strongly interconnected urban world need to be based on high value information extracted from multiple datasets by well-defined data preparation steps. To derive all necessary insights supporting the decision-making process, two technical components are essential: scalable and reliable IT infrastructure; and fast accessible, heterogeneous big datasets. DIAS data platforms offer all-in-one access to satellite imagery and high value services (CCS). The WEkEO platform was selected for CURE system development, as the EU's Copernicus DIAS reference service for environmental data, virtual environments for data processing and skilled user support. Moreover, it offers a wide range of pre-processed Sentinel data, as well as data from CCS or in-situ. Besides data availability, WEkEO offers additional computing infrastructure in terms of typical cloud virtualised engines. These are arranged along different virtual processing environments, fashioned to serve the required distributed processing system. The proactive involvement of users, as well as other societal representatives allied to an active co-creation process enables an effective and dynamic exchange ensuring that demands and needs are effectively addressed by CURE.

### CURE as knowledge driven platform

CURE has delivered an effective source of Copernicus-derived geo-information for urban planning and at the same time has enriched scientific knowledge with targeted spatial information required to support research enhancing resilience. A major scientific challenge of our times is to identify the contribution of urban environments to global climate change i.e., greenhouse gases and aerosol emissions, and urban temperature trends. Research to support the development of effective mitigation and adaptation strategy responses to heat wave events induced by climate change and urban heat island effects and associated mitigation solutions are critically important for future and emerging applications of CURE. The temporal dynamics of climate-related parameters in cities and their spatial extent can be derived from CCS through CURE, providing excellent information supporting the study of dependence on city structure and land-use changes. Furthermore, CURE results have the potential to support numerous research activities, for example: evaluation of the impacts of adaptation/mitigation strategies in cities, including NBS implementation (heat/CO_2_ emission, air quality, thermal comfort, health impact, flood risk products), bioclimatic rehabilitation of urban open spaces (NBS, heat emission products), urban metabolism (heat and CO_2_ emission products), local climate zoning (local scale surface temperature dynamics, heat storage products), thermal comfort studies (thermal comfort products, NBS), estimation of bioclimatic indicators (heat emission and storage products), epidemiologic studies related to the quantification of the effects of thermal stress on human health (thermal comfort, health impacts products), air quality studies (air quality and health impacts products), local and city scale energy efficiency studies (heat storage products) and urban vulnerability assessments at local scale (flood risk products, subsidence, movements and deformation products, NBS).

## Conclusions

CURE demonstrates potential as a relevant and timely tool helping policy-makers tracking local progress in the attainment of their resilience targets, by increasing the value of CCS for future emerging applications and in supporting the development of downstream services delivering urban resilience. Therefore, CURE is expected to promote more efficient urban planning delivering climate change mitigation and adaptation objectives (environmental benefit), resulting in improved thermal comfort and air quality (societal benefit), as well as an enhanced energy efficiency (economic benefit). CURE thereby supports strategies for resilience planning at local and city scales, by offering tools to cities for implementing the SDGs and the New Urban Agenda, as well as the EU Adaptation Strategy. Furthermore, CURE offers valuable resources supporting the implementation of the EU Missions: “Adaptation to Climate Change: support at least 150 European regions and communities to become climate resilient by 2030” and “100 climate-neutral and smart cities by 2030”. CURE thus exploits the European capacity for space-borne observations to enable the development of innovative services in the field of urban sustainability and resilience.

### Supplementary Information

Supplementary Figures.

## Data Availability

The datasets generated and/or analysed during the current study are available in: http://portal.cure-copernicus.eu, https://zenodo.org/communities/cure-h2020 and 10.5281/zenodo.6876179.
