# Supplementary material for: Copernicus for urban resilience in Europe
Source: Sci Rep. 2023 Sep 27;13:16251. doi: 10.1038/s41598-023-43371-9 (PMC10533551; doi:10.1038/s41598-023-43371-9)
Supplement: Supplementary file 1 — Supplementary Figures. [file 41598_2023_43371_MOESM1_ESM.pdf]

Copernicus for Urban Resilience in Europe

Nektarios Chrysoulakis[1], David Ludlow[2], Zina Mitraka[1], Giorgos Somarakis[1], Zaheer Khan[2], Dirk Lauwaet[3], Hans Hooyberghs[3], Efrén Feliu[4], Daniel Navarro[4], Christian Feigenwinter[5], Anne Holsten[6,11], Tomas Soukup[7], Mario Dohr[8], Mattia Marconcini[9] and Birgitte Holt Andersen[10]

[1] Foundation for Research and Technology Hellas, Institute of Applied and Computational Mathematics, Remote Sensing Lab, Heraklion, Greece
[2] University of the West of England, Bristol, United Kingdom
[3] Flemish Institute for Technological Research (VITO), Mol, Belgium
[4] TECNALIA, Basque Research and Technology Alliance (BRTA), Derio, Spain
[5] Universitaet Basel, Basel, Switzerland
[6] PIK, Potsdam Institut fuer Klimafolgenforschung, Potsdam, Germany
[7] Gisat S.R.O., Prague, Czech Republic
[8] GeoVille Informationssysteme und Datenverarbeitung GMBH, Innsbruck, Austria
[9] DLR, Deutsches Zentrum für Luft- und Raumfahrt, Weßling, Germany
[10] CWare Aps, Copenhagen, Denmark
[11] Bauhaus Earth, Berlin, Germany

The analysis of the functionality and the technological solutions of the CURE prototype is out of scope of this study, but a dedicated report on these issues has been developed and it is accessible via the CURE web-site (http://cure-copernicus.eu/deliverables.html).

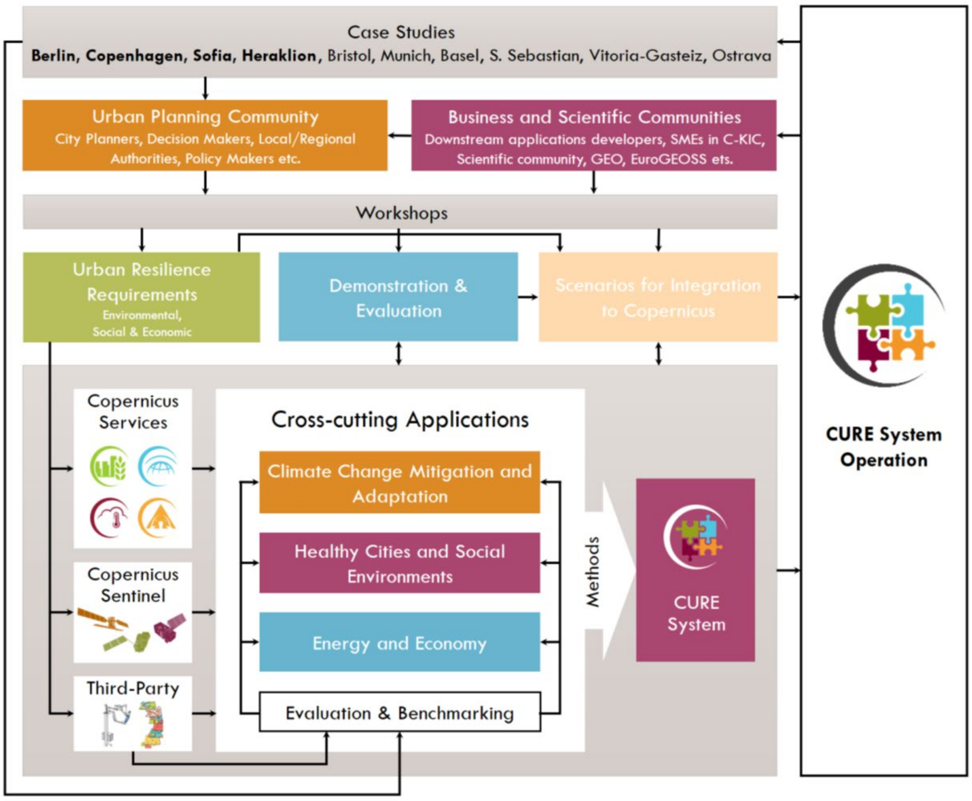

Fig. S1 | *Flowchart of the CURE methodological concept.*

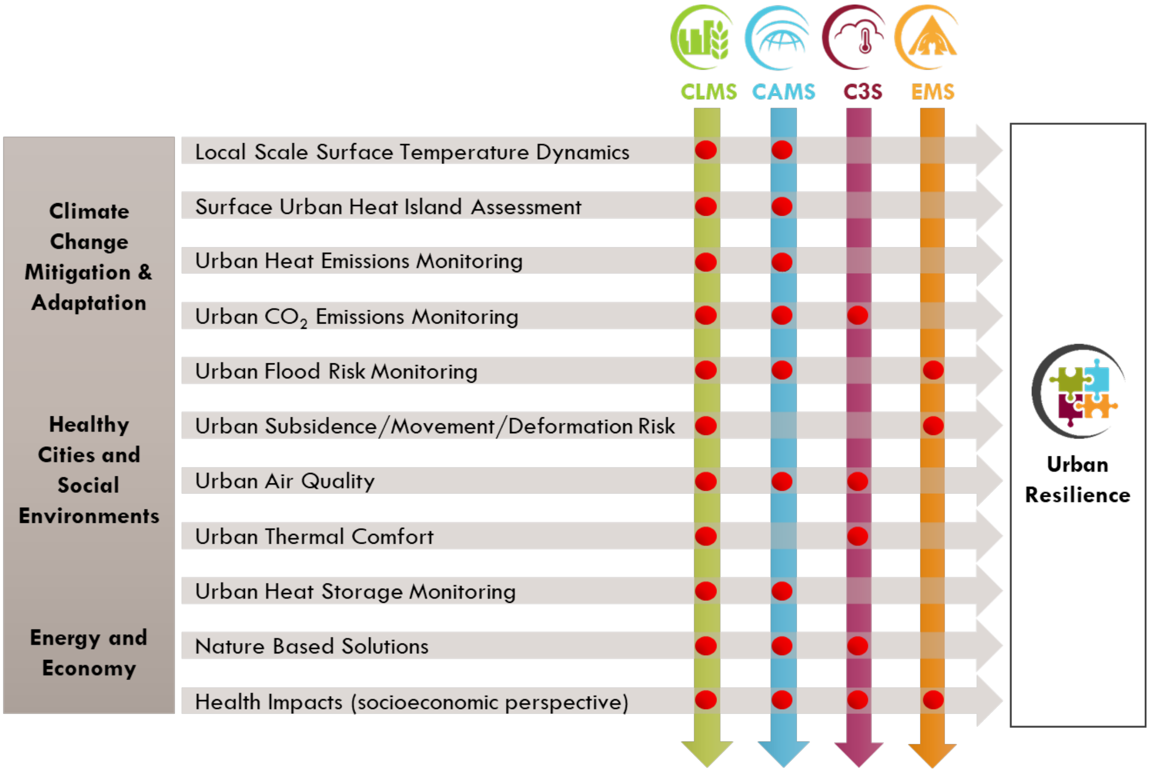

Fig. S2 | *The CURE applications development across four Copernicus Core Services, reflecting the urban sustainability dimensions shown at left, towards increasing urban resilience. Dots indicate which Core Service contributes to which cross-cutting application.*

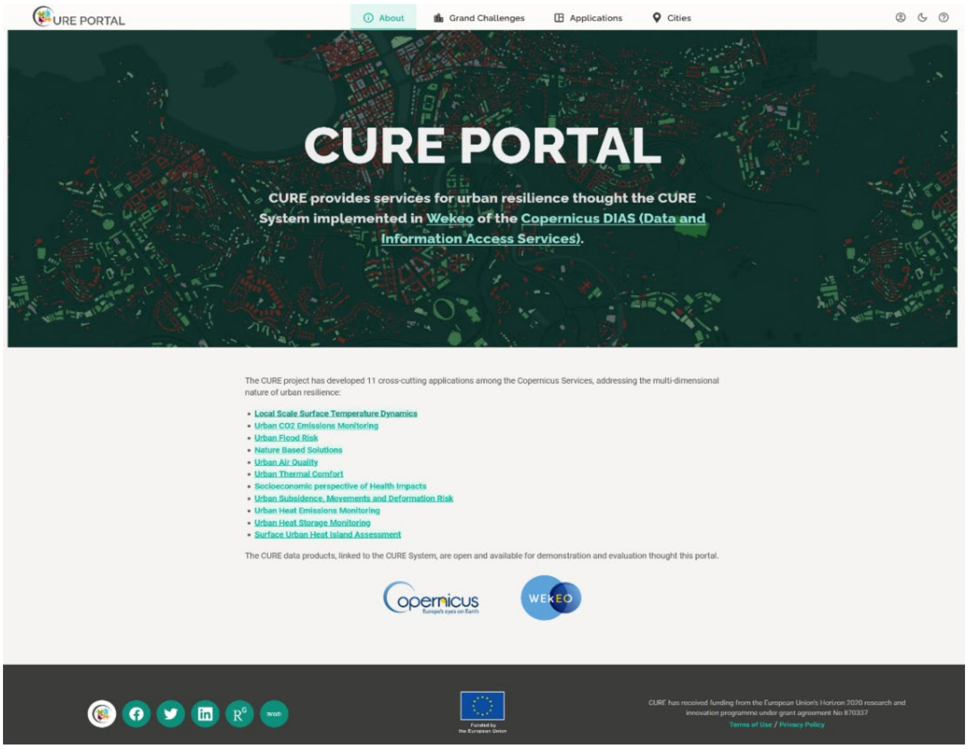

Fig. S3 | *The CURE portal providing access to the CURE System on DIAS at:* [*http://portal.cure-copernicus.eu*](http://portal.cure-copernicus.eu)

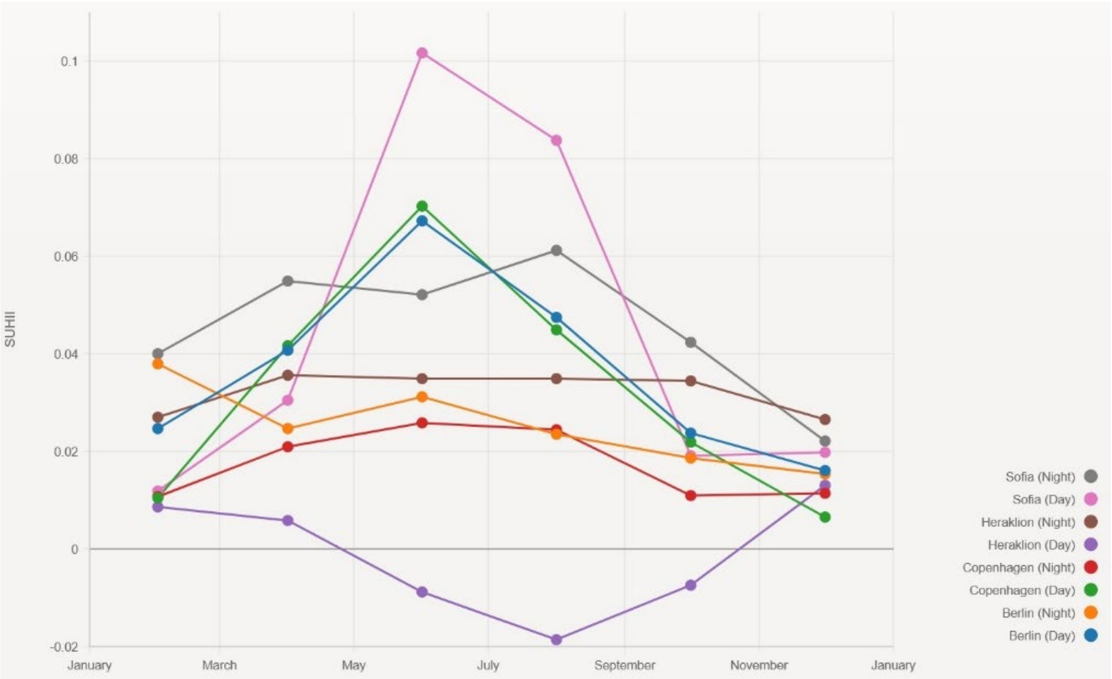

Fig. S4 | *SUHI profiles for CURE "front runner" cities (Sofia, Heraklion, Copenhagen and Berlin). Results for "front-runner" cities that are derived for bi-monthly LST data of the years 2018 and 2019 calculated via AP02.*

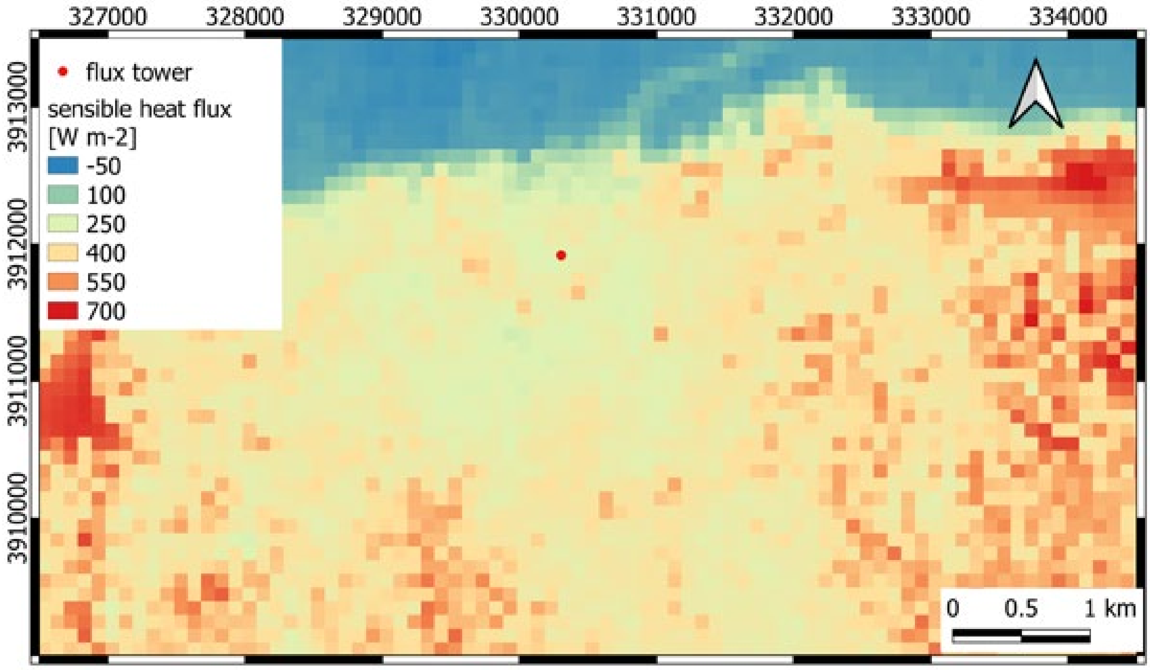

Fig. S5 | *Daytime spatial distribution of the turbulent sensible heat flux (Q_H) for the city of Heraklion. Map created with QGIS software, version 3.16 (www.qgis.org).*

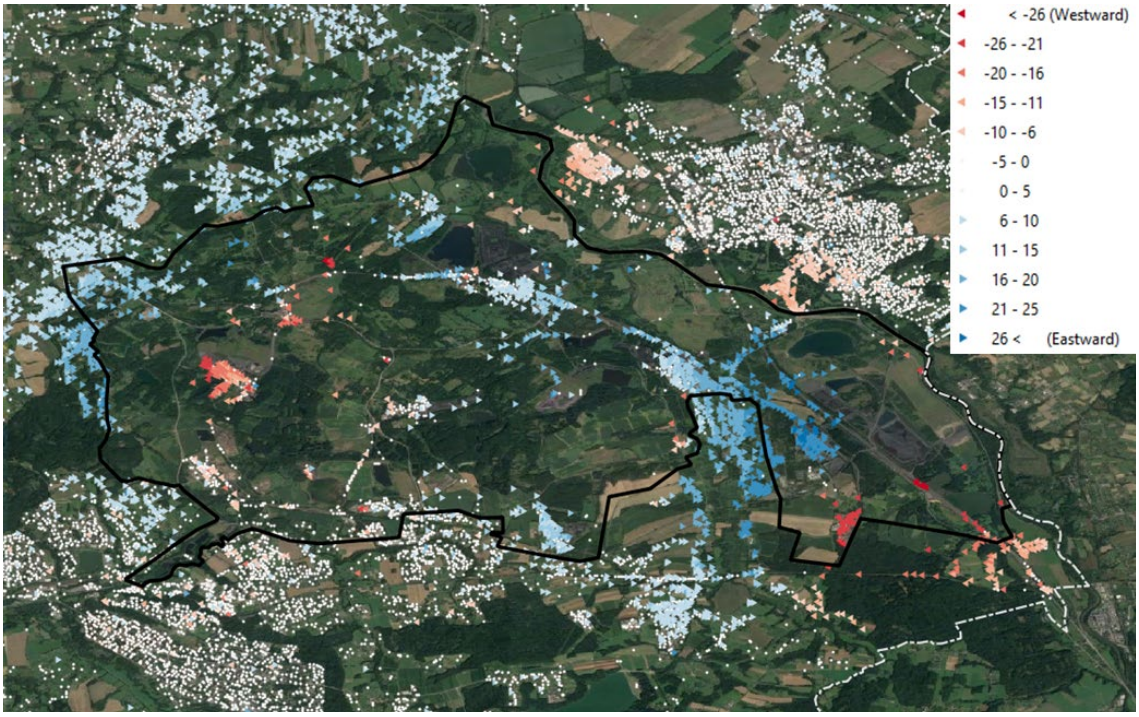

Fig. S6 | *Horizontal terrain movement for Poho mining area in Ostrava "follower city". Map created with QGIS software, version 3.16 (www.qgis.org).*

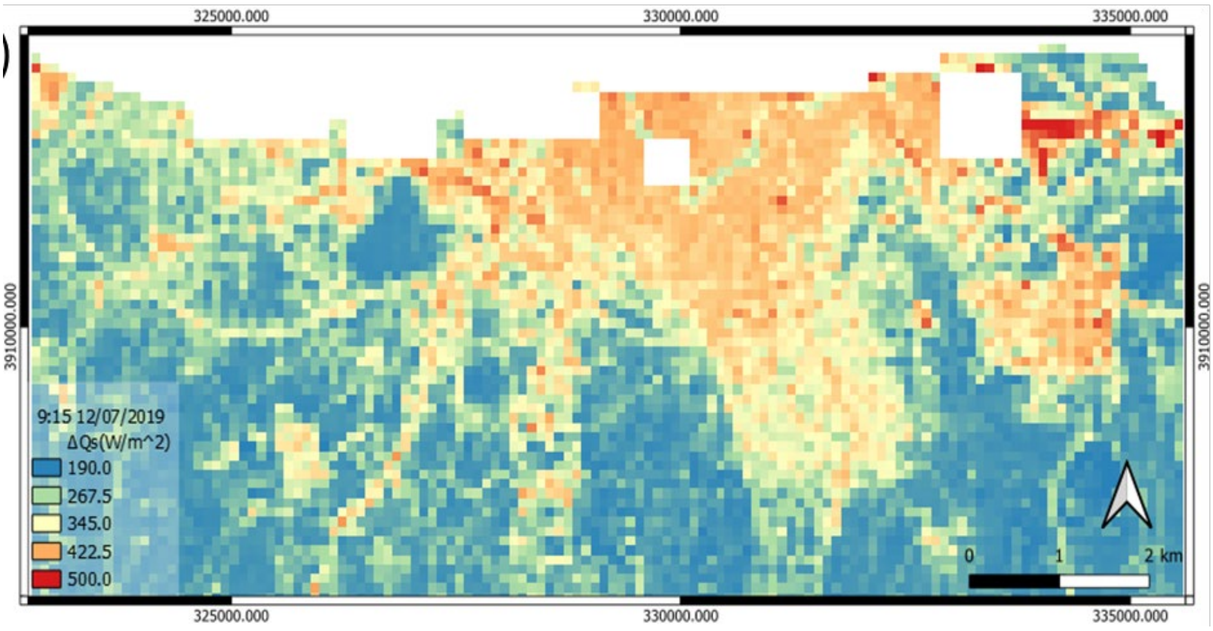

Fig. S7 | *Net change in heat storage (ΔQs) daytime spatial distribution (W m^{-2}) for Heraklion, on 21 July 2019, at 12.15 local time. The sea has been masked out, therefore the respective area appears in white. Missing values (also in white), i.e. in the coastline and some inside the images, are due to the cloud cover mask of Sentinel-3 imagery. Map created with QGIS software, version 3.16 (www.qgis.org).*

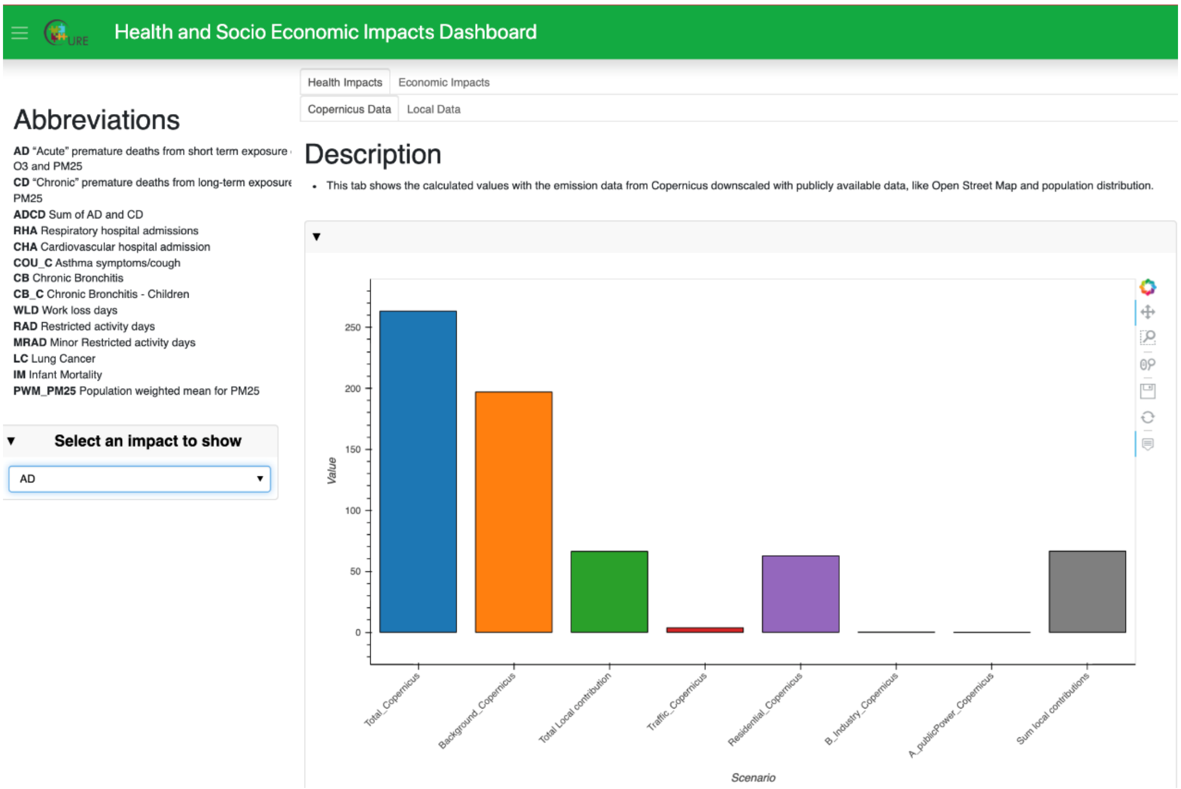

Fig. S8 | *Screenshot of the interface of health and economic costs of air pollution for Sofia: Acute Premature deaths (AD) based on Copernicus data (total, background, residential, traffic and industry, local).*
